# Monitoring liver transplant rates in persons diagnosed with hepatitis C: a data linkage study, England 2008 to 2017

**DOI:** 10.2807/1560-7917.ES.2019.24.41.1900176

**Published:** 2019-10-10

**Authors:** G Ireland, R Simmons, M Hickman, M Ramsay, C Sabin, S Mandal

**Affiliations:** 1 National Infection Service, Public Health England, London, United Kingdom; 2 The National Institute for Health Research Health Protection Research Unit (NIHR HPRU) in Blood Borne and Sexually Transmitted Infections at University College London, United Kingdom; 3 The National Institute for Health Research Health Protection Research Unit (NIHR HPRU) in Evaluation of Interventions at University of Bristol, Bristol, United Kingdom; 4 Population Health Sciences, Bristol Medical School, Bristol, United Kingdom; 5 University College London, London, United Kingdom

**Keywords:** hepatitis C, hepatitis C virus, HCV, liver transplantation, direct‐acting antivirals, England, blood-borne infections, laboratory surveillance, epidemiology

## Abstract

**Introduction:**

Liver transplantation is an important measure of burden from hepatitis C virus (HCV)-associated liver disease.

**Aims:**

To describe transplant rates and survival in individuals with HCV infection from 2008 to 2017 in England through data linkage.

**Methods:**

This is a retrospective observational cohort study. Laboratory reports of HCV infection were linked to the Liver Transplant Registry for individuals aged 15 years and over, first diagnosed between 1998 and 2017. We estimated age-sex standardised incidence rates and used Poisson regression to investigate predictors of liver transplantation and test for a change in incidence after introduction of direct-acting antivirals (DAAs) in 2014. Kaplan-Meier survival analysis was used to calculate post-transplant survival rates.

**Results:**

Of 124,238 individuals diagnosed with HCV infection, 1,480 were registered and 1,217 received a liver transplant. Of individuals registered, 1,395 had post-HCV cirrhosis and 636 had hepatocellular carcinoma (618 also had post-HCV cirrhosis). Median time from HCV diagnosis to transplant was 3.4 years (interquartile range: 1.3–6.8 years). Liver transplant rates were lower 2014–17 compared with 2011–13 (incidence rate ratio: 0.64; 95% confidence interval: 0.55–0.76). Survival rates were 93.4%, 79.9% and 67.9% at 1, 5 and 10 years, respectively. Data linkage showed minimal under-reporting of HCV in the transplant registry.

**Conclusion:**

In the post-DAA era, liver transplant rates have fallen in individuals with HCV infection, showing early impact of HCV treatment scale-up; but the short time from HCV diagnosis to liver transplant suggests late diagnosis is a problem.

## Introduction

In 2018, around 113,000 individuals were estimated to be chronically infected with hepatitis C virus (HCV) in England [1]. These individuals are at increased risk of cirrhotic end-stage liver disease (ESLD) and hepatocellular carcinoma (HCC), for which mortality rates had been increasing – doubling over 10 years from 2005 to 2014 – until recent years [2]. Liver transplantation may be indicated if liver decompensation deteriorates. Between 2008 and 2014 in England, an average of 134 individuals with post-HCV cirrhosis were registered for a liver transplant and 108 received a liver transplant each year, accounting for 17–21% of all liver transplants [2].

HCV-related ESLD is preventable with early diagnosis and treatment of HCV infection, but historically estimated treatment levels in the United Kingdom (UK) have been low (ca 3% annually) [3]. Low treatment rates have been attributed to: (i) interferon-based regimens which required injections, (ii) long treatment durations, (iii) poor tolerability and (iv) moderate efficacy, as defined by a sustained virological response (SVR). In comparison, the newly introduced direct-acting antivirals (DAAs) are taken orally, have short regimens and have SVRs that are above 95% [4]. Since 2015, National Health Service (NHS) England has rolled out DAAs in a managed care programme with annual scale-up in treatment slots; an estimated 24,592 people had been treated between the financial years of 2015/16 and 2017/18 [5]. Widespread treatment with DAAs not only prevents the development of severe liver disease in individuals with HCV but can also improve liver function in individuals with advanced liver disease, which should contribute to a fall in the burden of HCV-associated disease and the need for liver transplantation [4,6,7].

Since the expansion of treatment with DAAs in England, a 43% drop in the number of liver transplant registrations has been recorded for individuals with post-HCV cirrhosis [2]. Vaziri et al. reported that the proportion of liver transplants attributed to HCV-associated cirrhosis fell from 10.5% to 4.7% between 2013 and 2016 and the proportion of liver transplants for cancer-associated HCV fell from 46.4% to 33.7% over the same period [8]. Similar results have been found in Italy, the United States (US) and Argentina [9-11]. A declining contribution of HCV-associated cirrhosis and cancer to transplants is helpful but is not a substitute for monitoring rates of HCV-associated transplants and may be limited by under-reporting of HCV coding in the liver transplant dataset, as has been observed in death registry data [12].

Through data linkage of routine laboratory reports of HCV and the NHS Blood and Transplant Service (NHSBT) liver transplant registry, we estimate liver transplant incidence rates and survival in the pre- (2008–13) and post-DAA (2014–17) eras, describe the characteristics of individuals who are registered for and underwent a liver transplant (2008–17) and explore any under-reporting of HCV in the transplant registry. We hypothesise that a reduction in transplant rates post introduction of DAAs will be observed, indicative of the early impact of DAA on HCV burden.

## Methods

Using data linkage, we conducted a retrospective observational cohort study to describe the characteristics of individuals diagnosed with HCV infection in England between 1998 and 2017, who were registered for and had received a liver transplant, and estimate liver transplant rates between 2008 and 2017.

### Data sources

#### Routine laboratory reports of HCV infection

HCV diagnoses were obtained from routine laboratory reports of HCV, defined as the detection of HCV antibody (anti-HCV) or HCV RNA in blood, submitted by English virology laboratories to Public Health England (PHE). The laboratory surveillance system does not distinguish between anti-HCV or HCV RNA positive individuals, so laboratory ‘confirmed’ cases are a mix of current and ever infected individuals. Laboratory HCV reports have been submitted to PHE (and its predecessor organisations) through paper forms or electronically since 1990 but laboratory reporting of notifiable organisms became mandatory in 2010. Reports include basic demographics (name, date of birth, sex and NHS number (a unique identifier for all individuals registered with the NHS within England)) and variable amounts of risk factor information (e.g. History of injecting drug use, testing within prison services, infection as a result of tattoos, piercings or blood products). Ethnicity is poorly recorded.

For this study, the routine laboratory reporting dataset was enhanced with information from additional sources. As the date of the first HCV diagnosis is critical, this was updated with information from the Sentinel Surveillance of Blood Borne Virus Testing (SSBBV) and Hospital Episode Statistics (HES) when possible. Established in 2002, SSBBV collects information on hepatitis A-E, HIV and HTLV tests (regardless of the result) from 23 participating sentinel laboratories in England [13,14]. Earlier diagnosis dates were identified in HES where an earlier inpatient stay was recorded with HCV ICD-10 diagnosis codes (B17.1 and B18.2). HCV treatment with DAAs (date and outcome) was obtained through linkage with the national HCV treatment monitoring and outcomes dataset, established in 2015. Date of death was obtained from the NHS Spine Patient Demographic Service.

Name, date of birth, sex and NHS number were used to de-duplicate reports. Information on individuals first diagnosed with HCV infection between 1998 and 2017 with sufficient identifiers for linkage (name, sex, date of birth and NHS number) were extracted from the database.

#### National Transplant Database

Information on individuals registered for a liver transplant in the National Transplant Database was obtained from NHSBT and included all liver transplant registrations between 1994 and 2017, followed up to May 2018. The dataset contains basic demographics (date of birth, sex and ethnicity) of the recipient, liver disease at registration and transplant, results of pre-transplant HCV tests and outcome of registration. Additional clinical information for individuals who received a liver transplant includes the UK Model for End-Stage Liver Disease (UKELD) score, encephalopathy grade and the presence of ascites and oesophageal varices. Registration on the transplant list can be considered elective (routine) or super-urgent (emergency). Outcome of registration includes: (i) still waiting for a transplant, (ii) transplant received, (iii) suspension from the registry (a temporary period of time during which a patient will not be considered for transplant e.g. if a patient was unfit or otherwise unavailable for transplant, (iv) death before transplant, and (v) removal from the registry if they no longer require a liver transplant. The transplant registry links with the Office for National Statistics (ONS) and is notified of the deaths of individuals who are on the waiting list, providing year and cause of death.

### Data linkage

The two datasets were linked deterministically using a combination of name, date of birth, sex and NHS number.

### Analysis

Age-sex standardised incidence rates (ASRs) of first liver transplant and first HCC-associated liver transplant were calculated for individuals aged 15 years and older who were diagnosed with HCV infection between 1998 and 2017 and are presented for 2008 to 2017. ASRs for 1998–2007 are not presented due to unstable rates and wide 95% confidence intervals (CI) (calculated using Poisson distribution) resulting from the smaller cohort size and limited follow-up time. Individuals who received a liver transplant before or within 6 months of HCV diagnosis were excluded from the analysis, as there was potential bias towards higher rates of diagnosis in individuals with major morbidity and in individuals who received a transplant before their reported HCV diagnosis were likely to have had a diagnosis prior to transplant which had not been reported to PHE. As a result, follow-up began 6 months after diagnosis and ended when the individual had a liver transplant, died or 31 December 2017 whichever occurred first. ASRs were also calculated for all liver transplants in England using ONS mid-year population estimates [15] and NHSBT data on all first liver transplants in England.

Individuals were classified as having a record of HCV on the liver transplant registry if they had ‘post HCV cirrhosis’, a positive anti-HCV or positive HCV RNA test. Alcohol-associated comorbidities were recorded if liver disease at registration or transplant included ‘alcoholic cirrhosis’. Individuals were recorded as dead if they had a date or year of death in either dataset.

STATA SE version 13 (Stata Corp, College Station, Texas, US) was used for statistical analysis. Wilcoxon rank-sum tests were used to compare continuous variables. Excluding individuals diagnosed with HCV following a liver transplant, Kaplan-Meier analysis was used to estimate the cumulative proportion of individuals having had a liver transplant at 1, 5 and 10 years after HCV diagnosis. Poisson regression was used to identify predictors of receiving a liver transplant in individuals diagnosed with HCV between 1998 and 2016 and to test for a change in incidence pre and post 2014. Factors included in these models were sex, age at diagnosis and calendar year (grouped as 1998–2001, 2002–04, 2005–07, 2008–10, 2011–13 and 2014–17). Individuals who received a liver transplant within 6 months of HCV diagnosis were excluded from this analysis and follow-up was calculated in the same way as for the ASRs. Kaplan-Meier survival analysis was used to calculate 1, 5 and 10-year post-transplant survival rates.

### Ethical statement

The PHE Caldicott Guardian approved the collection and processing of confidential patient data, without patient consent, from routine laboratory reports of HCV infection, SSBBV and for linkage of these laboratory data to the liver transplant registry under Regulation 3 of the Health Service (Control of Patient Information) Regulations 2002. Regulation 3 allows the processing of patient information for recognition, control and prevention of communicable disease and other risks to public health.

## Results

Of 161,820 individuals aged 15 years and older diagnosed with HCV in England between 1998 and 2017, 124,238 (76.8%) had sufficient identifiers for linkage with the liver transplant registry. Where reported, 84,444 (68.5%) of all individuals with HCV were male and the median age at report was 39 years (interquartile range (IQR): 31–48 years) (Table 1). Of those with confirmed HCV, 1,480 (1.2%) were matched to the liver transplant registry and 15,282 (12.3%) had received HCV treatment with DAAs. The cumulative proportion of individuals who had received a liver transplant was 0.2%, 0.7% and 1.1% at 1, 5 and 10 years after HCV diagnosis, respectively.

**Table 1:** Characteristics of individuals diagnosed with hepatitis C virus, by transplantation status, England, 1998–2017

Characteristics	HCV diagnoses	Registered		Transplanted	
		n	%	n	%
**Total**	**124,238**	**1,480**	**1.2**	**1,217**	**1.0**
**Sex**					
Male	84,444	1,170	1.4	959	1.1
Female	38,920	310	0.8	258	0.7
Not reported	874	0	0.0	0	0.0
**Age (years)**					
15–29	24,231	23	0.1	20	0.1
30–39	40,398	172	0.4	138	0.3
40–49	31,711	578	1.8	474	1.5
≥ 50	27,688	707	2.6	585	2.1
Unknown	210	0	0.0	0	0.0
**Year of Diagnosis**					
1998–02	17,124	464	2.7	392	2.3
2003–07	32,908	490	1.5	402	1.2
2008–12	36,648	406	1.1	320	0.9
2013–17	37,558	120	0.3	103	0.3
**Region**					
East Midlands	6,833	43	0.6	35	0.5
East of England	9,940	213	2.1	185	1.9
London	27,103	317	1.2	268	1.0
North East	3,513	35	1.0	31	0.9
North West	24,454	215	0.9	151	0.6
South East	12,564	163	1.3	138	1.1
South West	13,366	138	1.0	114	0.9
West Midlands	11,129	201	1.8	171	1.5
Yorkshire and The Humber	15,248	155	1.0	124	0.8
Unknown	88	0.0	0.0	0.0	0.0

HCV: hepatitis C virus.

### Transplant registry

Of 1,480 individuals registered for a liver transplant, 79.1% were male and the median age at registration was 53 years (IQR 48–58 years) (Table 2). Among registrants, 94.3% had post-HCV cirrhosis, 43.0% had a record of HCC (618 of whom also had post-HCV cirrhosis), 1,187 (80.2%) had a positive HCV antibody or RNA test recorded on the liver transplant registry and 25.9% had a record of alcoholic cirrhosis. Only 47 (3.2%) of linked transplant registrants had no clinical indicators of a history of HCV infection (indicated by post-HCV cirrhosis or a positive HCV test).

**Table 2:** Demographic and clinical characteristics of persons diagnosed with hepatitis C virus and registered for a liver transplant (n = 1,480) and received a liver transplant (n = 1,217), England, 1998–2017

Characteristics	n	%
**Total registered for liver transplant**	**1,480**	**100**
**Sex^a^**		
Male	1,170	79.1
Female	310	20.9
**Ethnicity^a^**		
White	1,159	78.3
Asian	240	16.2
Black	39	2.6
Other	42	2.8
**Urgency of registration^a^**		
Elective	1,464	98.9
Super urgent	16	1.1
**Disease at registration^a,b^**		
HCC	636	43.0
post-HCV cirrhosis	1,395	94.3
Alcoholic cirrhosis	383	25.9
**Transplant outcome**		
Transplanted	1,217	82.2
Died	94	6.4
Removed from registry	166	11.2
Awaiting transplant	3	0.2
**Total received a liver transplant**	**1,217**	**100**
**UKELD score at transplant**		
Median	52.5 (49–56)	
**Ascites at transplant**		
Yes	582	47.8
**Encephalopathy grade at transplant**		
No encephalopathy	916	76.0
Grade 1	212	17.6
Grade 2	58	4.8
Grade 3	10	0.8
Grade 4	9	0.7
Not reported	12	
**Oesophageal varices at transplant**		
No previous variceal bleeding	417	35.0
Previous variceal bleeding	293	24.6
Not present	481	40.4
Not reported	26	
**Multiple liver transplants**		
2	79	6.5
3	3	0.2

HCC: hepatocellular carcinoma; HCV: hepatitis C virus; UKELD: United Kingdom Model for End-Stage Liver Disease.

^a^ At registration for liver transplant.

^b^ Three diseases at registration can be listed so some people may have been counted twice.

As at July 2018, 1,217 of the 1,480 (82.2%) registered individuals had received a liver transplant; 13 transplants were from living donors, 94 (6.4%) died before receiving a transplant, 116 (11.2%) were removed from the registry (including two who were suspended from the registry) and three (0.2%) remained on the registry on the follow-up date (Table 2). Persons who died while on the registry were on it for a median of 80 days (IQR: 25–158 days).

### Received a liver transplant

Of 1,217 individuals who received a transplant, the median age at transplant was 53 years (IQR 48–58) and individuals with a record of alcoholic cirrhosis were transplanted at a younger median age than those with no record of alcoholic cirrhosis (51 vs 54 years respectively, p < 0.0001). Individuals waited for a median of 75 days (IQR: 28–174 days) between registration and transplant and the majority (1,155; 94.9%) received their transplant after or on the same day as their HCV diagnosis in routine laboratory reporting (median: 3.4 years; IQR: 1.3–6.8 years), while 66 individuals (5.1%) persons received a transplant before an HCV diagnosis in routine laboratory reporting (range: 1 day–20.0 years). At time of the first transplant, the median UKELD score was 52.5 (IQR: 49–56), 47.8% had ascites and (where reported) the majority did not have encephalopathy (76.0%) or oesophageal varices (40.4%) (Table 2).

**Figure 1:**
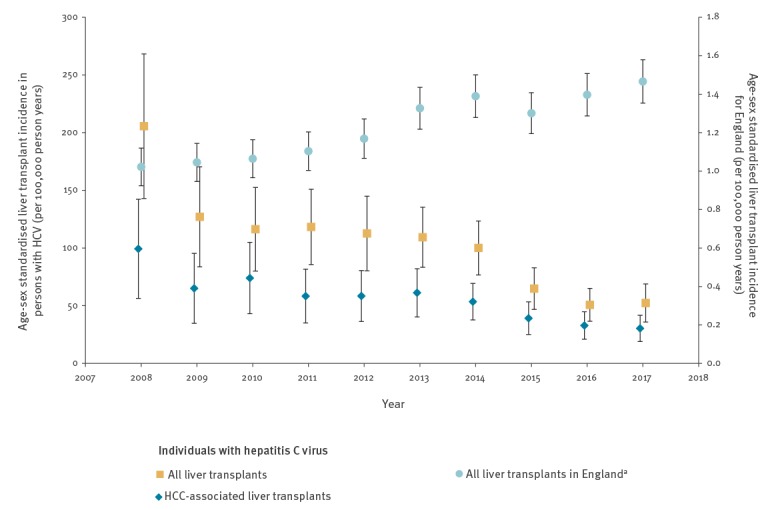
Age-sex standardised incidence rates by liver transplant and HCC-associated liver transplant in individuals diagnosed with hepatitis C virus between 1998–2017, and by all liver transplants in England, 2008 and 2017

HCC: hepatocellular carcinoma; HCV: hepatitis C virus.

^a^ All liver transplants in England are irrespective of HCV diagnosis and cause.

Age-sex standardised liver transplant incidence and HCC-associated liver transplant incidence between 2008 and 2017 are presented in Figure 1. ASRs indicate that rates for both were stable between 2008 and 2014 but fell between 2015 and 2017. Using Poisson regression, incidence rate ratios (IRR) for liver transplant were higher in males (IRR: 2.01; 95% CI: 1.73–2.34) and older persons at HCV diagnosis (per 10-year increase, IRR: 1.76; 95% CI: 1.69–1.83). When compared with 2011–13, the IRRs for 1998–2001 and 2002–04 were higher (IRR: 2.42, 95% CI: 1.79–3.26 and IRR: 1.76, 95% CI: 1.40–2.21, respectively), rates for 2005–07 and 2008–10 were similar (IRR: 0.83; 95% CI: 0.66–1.05 and IRR: 1.07; 95% CI: 0.89–1.29, respectively) and rates for 2014–17 were lower (IRR: 0.64; 95% CI: 0.55–0.76). These trends are in contrast to those seen ASRs in England as a whole (irrespective of HCV diagnosis and cause) which showed an increasing trend between 2008 and 2017 (Figure 1).

### Survival following a liver transplant

**Figure 2:**
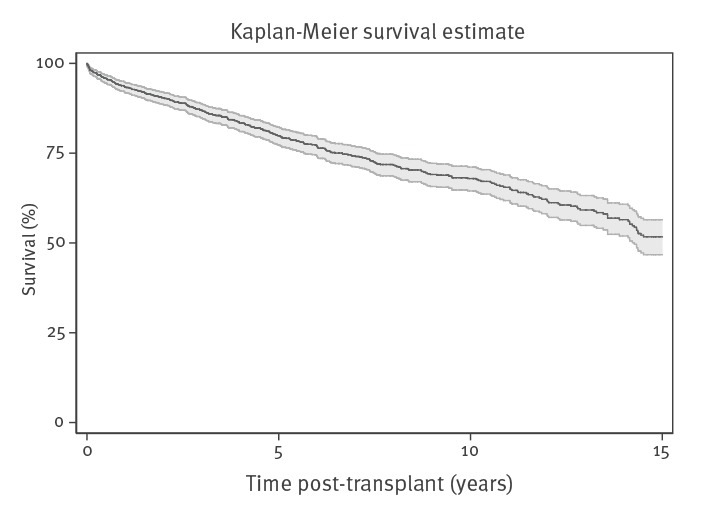
Kaplan-Meier survival curve for individuals with hepatitis C virus infection who received a liver transplant, England, 1998–2018

As at 30 May 2018, 348 (29.1%) of those who received a transplant had died, a median of 3.6 years after their first transplant (IQR 1.2–7.4 years). Where the cause of death was available (249/366; 71.6%), the most common cause of death was ‘multi-system failure’ (45; 18.1%), followed by ‘other identified cause of death’ (31; 12.4%). Using the Kaplan-Meier method, unadjusted survival rates following a liver transplant were 93.4% (95% CI: 91.8–94.6), 79.9% (95% CI: 77.3–82.2) and 67.9% (95% CI: 64.5–71.1) at 1, 5 and 10 years (Figure 2).

## Discussion

Among persons diagnosed with HCV between 1998 and 2017, 1.2% were registered for a liver transplant, 82.2% of whom received a transplant. Liver transplant rates were higher in males and in those who were older at HCV diagnosis and lower between 2014 and 2017, the post-DAA era, when compared with 2011–13. The majority of registrants had evidence of post-HCV cirrhosis and/or a record of HCC, with one in four having a record of alcoholic cirrhosis. In individuals who received a transplant, the median time between HCV diagnosis and a transplant was 3.4 years and 69.7% of persons who received a transplant survived 10 years. Data linkage revealed that under-reporting of HCV diagnosis in the transplant registry was not a major concern.

### Limitations

There are several limitations of this study. First, it is possible that individuals were diagnosed with HCV before PHE was notified or their diagnosis was not reported to PHE at all, thus impacting follow-up time. This would be more of an issue for individuals diagnosed before 2010, as since then, laboratories are legally required to notify PHE of all positive HCV tests. However, individuals, who were diagnosed before 2010 (pre-legislation) may also have been tested with their HCV diagnosis reported post-2010 (post-legislation) and therefore captured in later years. Linkage with other surveillance systems can be used to update diagnoses dates before 2010. Second, we did not have any information on whether individuals were acutely or chronically infected with HCV as this information cannot be derived from the laboratory reports. However, as individuals with chronic infection are more likely to progress to advanced liver disease, we can assume that most individuals who were registered for a liver transplant had chronic hepatitis rather than acute fulminant hepatitis type presentation. Third, we had no information on the stage of liver disease at diagnosis in the HCV laboratory reports. This information would have been useful, as we found short time lag between HCV diagnosis and transplant registration, which we can only speculate is due to late diagnosis of liver disease and/or HCV when disease stage is already advanced. If the disease stage was available, we could have investigated liver transplant rates by disease stage at presentation.

### Other evidence and implications

Through linked data, we identified a decline in HCV-associated liver transplant rates after 2014, consistent with unlinked published data for England [2,8] and internationally [9-11]. A separate study within the UK [8], using unlinked data, reported registered transplants for patients with HCV-related cirrhosis fell from 10.5% in 2013 to 4.7% in 2016. In Italy, a decrease was observed between pre-DAA and post-DAA periods for transplant waiting lists overall as well as among patients among those with decompensated cirrhosis, whereas figures associated with HCC remained stable [10]. A downward trend was identified in liver transplants among patients with decompensated cirrhosis in Argentina [11]. A decline has also been reported in the US [9] among patients waiting for a transplant, with a lower likelihood of being on the waiting list in the DAA era compared with the pre-DAA era (Hazard Ratio 0.83). The decrease in liver transplant incidence in England among individuals with HCV within this study contrasts with the trend observed for all liver transplants, where rates have increased in recent years, albeit on a much smaller scale. It is encouraging to see a reduction in HCV-associated liver transplant rates and we hope that the incidence will continue to fall as case-finding activity increases helping to reduce the undiagnosed burden and ensuring that more individuals are treated with DAAs. A similar, although non-significant, decline has also been found in age-sex standardised liver mortality rates among anti-HCV positive individuals [16].

Time from HCV diagnosis to transplant was short. Mindful of the established natural history of HCV infection causing liver disease progression over several decades [17], this indicates that this linked cohort of individuals with HCV was diagnosed late. As a result, individuals are likely to present with advanced liver disease, which is less amenable to HCV cure and may progress despite HCV cure. Poorer response rates to HCV treatment among patients with cirrhosis have been reported [18-20]. In an Italian study, the risk of failing SVR12 was 5.39 times higher among patients with decompensated cirrhosis and 1.56 times with compensated cirrhosis compared with those without cirrhosis [19]. In an American study, the efficacy was found to be significantly reduced to 73% and 91% among those with decompensated and compensated cirrhosis, respectively [20]. Two further studies, one European and Canadian-based and the other an Italian study, observed that the risk of liver disease continued among patients with cirrhosis despite SVR [21,22]. The linkage between SSBBV and the ONS death registry corroborate this finding, as the median time from HCV diagnosis to death (any cause) was 3 years [16]. While there are limited population-level data in England currently on disease stage at time of diagnosis of HCV, data from the national HCV treatment monitoring dataset indicate that around 40% of individuals who have received treatment (2015–18) had moderate fibrosis to decompensated cirrhosis at the time of the decision to proceed with treatment. This is in part due to NHS England’s phased implementation of DAA treatments, prioritising patients with advanced cirrhosis and fibrosis before expanding treatment access to patients with milder disease.

Our results from the survival analysis following a transplantation in an individual with an HCV diagnosis (1 year: 93.4%; 5 years: 79.9%; 10 years: 67.9%) are similar to those found by Su Yin Lau et al. (1 year: 95.2%; 5 years: 78.2%) and for all individuals who received a liver transplant in the UK (1 year: 93.4%; 5 years: 80.5%) [23,24]. It is encouraging that survival following liver transplantation in individuals with HCV is similar to all persons receiving a liver transplant and that HCV does not appear to have an additive negative impact on survival rates.

It is important to note that under-reporting of HCV-associated transplants is minimal, with only 3.2% of linked individuals having no record of HCV-associated disease or positive HCV tests in the transplant registry. This is much lower than the under-reporting with HCV-associated mortality in England, where 41% of persons who died of liver-related causes linked to an HCV laboratory diagnosis did not have HCV recorded as a contributory cause in the deaths registry [12]. A similar level of under-reporting in mortality was also identified in the US [25] and Scotland [26], with 41% and 52% of individuals who died of liver-related causes, respectively, having HCV indicated on their death certificate. The low level of under-reporting is important to quantify as an accurate recording of HCV in transplant registry data reduces uncertainty in modelling to estimate prevalence and disease burden. These results improve confidence in the use of unlinked HCV-associated liver transplant data as a metric for monitoring progress towards elimination goals in England. Data linkage of HCV diagnoses to a transplant registry also offers an easily reproducible method for other European countries whose surveillance systems are limited by under-reporting of HCV diagnoses in transplant databases and/or lack of information on long-term health outcomes in HCV laboratory diagnoses datasets. Data linkage also allows the burden of disease in the HCV diagnosed population, to be better understood and for an individual in this population to be followed from diagnosis to outcome. This is important when comparing this group to individuals who did not receive a transplant and for understanding these data in the context of a country’s HCV epidemic. Liver transplantation, associated with chronic HCV infection, is an important measure of liver disease from HCV infection and countries may, therefore, choose to monitor this metric as an additional indicator (similar to HCV-related mortality and new chronic infections) of the impact of new DAA treatments and their contribution towards the WHO elimination goal [27].

### Conclusion

By linking routinely collected datasets, we found 1.2% of persons with HCV were registered for a liver transplant, with higher rates in males. The short median time from diagnosis to transplant suggests missed opportunities for earlier diagnosis before irreversible liver damage has occurred. Although based on early data, our analyses suggest that transplant rates have decreased further since 2014 (the year that DAAs became available in England). Efforts to reduce the undiagnosed HCV burden by active case finding, earlier diagnosis, improved engagement in care and expanded DAA treatment to people with mild or no fibrotic liver disease, could lead to sustained and potentially accelerated reductions in HCV-associated liver transplants as progression to cirrhotic liver disease will become less common. Our study, therefore, provides a baseline from which to benchmark changes in HCV-associated transplant rates as part of evaluating the impact of DAAs and other interventions on eliminating HCV as a global public health threat by 2030.
